# Knowledge of the Disease, Perceived Social Support, and Cognitive Appraisals in Women with Urinary Incontinence

**DOI:** 10.1155/2016/3694792

**Published:** 2016-12-21

**Authors:** Katarzyna Szymona-Pałkowska, Konrad Janowski, Agnieszka Pedrycz, Dariusz Mucha, Tadeusz Ambroży, Piotr Siermontowski, Jolanta Adamczuk, Marta Sapalska, Dawid Mucha, Janusz Kraczkowski

**Affiliations:** ^1^Department of Clinical Psychology, John Paul II Catholic University of Lublin (KUL), Lublin, Poland; ^2^Faculty of Psychology, University of Finance and Management in Warsaw, Warsaw, Poland; ^3^Department of Histology and Embryology, Medical University of Lublin, Lublin, Poland; ^4^Department of Physical Education and Sport, Academy of Physical Education, Krakow, Poland; ^5^Department of Maritime & Hyperbaric Medicine, Military Institute of Medicine, Gdynia, Poland; ^6^SP ZOZ Nałęczów (Private Practice), Nałęczów, Poland; ^7^Social Insurance Institution (ZUS), Lublin, Poland; ^8^Department of Obstetrics and Pathology of Pregnancy, Medical University of Lublin, Lublin, Poland

## Abstract

Social support and knowledge of the disease have been shown to facilitate adaptation to a chronic disease. However, the adaptation process is not fully understood. We hypothesized that these factors can contribute to better adaptation to the disease through their impact on disease-related cognitive appraisal. To analyze the links between social support and the knowledge of the disease, on one hand, and disease-related appraisals, on the other hand, one hundred fifty-eight women with stress UI, aged 32 to 79, took part in the study. Questionnaire measures of knowledge of UI, social support, and disease-related appraisals were used in the study. The level of knowledge correlated significantly negatively with the appraisal of the disease as Harm. The global level of social support correlated significantly positively with three disease-related appraisals: Profit, Challenge, and Value. Four subgroups of patients with different constellations of social support and knowledge of the disease were identified in cluster analysis and were demonstrated to differ significantly on four disease-related appraisals: Profit, Challenge, Harm, and Value. Different cognitive appraisals of UI may be specifically related to social support and knowledge of the disease, with social support affective positive disease-related appraisals, and the knowledge affecting the appraisal of Harm.

## 1. Introduction

Urinary incontinence (UI) is defined as involuntary passage of urine through the urethra with the symptoms depending on the type of the underlying disease. UI is one of the most widespread chronic diseases which poses a serious social problem. Based on its symptoms and causes, the Standardization Committee of the International Continence Society distinguishes the following types of UI: stress incontinence, urge incontinence, mixed incontinence, overflow incontinence, and neurological incontinence [1–3]. Epidemiological data suggest that UI symptoms occur worldwide in 4–10% of women in their twenties, in as many as 60% of women in their sixties, and in 70–80% of women over 65 [1, 4–12]. Epidemiological data reveal that over 40% of women will experience UI symptoms during their lifetime [13, 14]. Most of them do not perceive the symptoms as indicators of a disease but see them as a physiological consequence of pregnancies and childbirths or simply as a natural manifestation of ageing [15]. The topic of urinary incontinence, usually regarded as shameful, has begun to appear in mass media in recent years, but mainly in the context of care and pharmacological products; however, prevention and forms of assistance that UI sufferers should be provided with are not sufficiently presented. Literature reveals that women take very long to report their UI symptoms. Extensive and interesting research was conducted by Kinchen et al. [16], covering 1970 women who were asked about their symptoms and a consultation with a medical professional concerning UI and its treatment was provided. The study demonstrated that over 50% of the female sufferers did not decide to report the symptoms or talk to a physician about them. The decision to seek treatment depended upon the duration of the symptoms, subjective perception of their severity, and a conviction that the symptoms might have been noticed by others. Very often, individuals with UI experience a psychological barrier in making use of social support due to an embarrassing nature of the symptoms [2, 17]. Other data [18] indicate that the general public is underinformed about UI.

Knowledge of the disease involves a range of beliefs based on the information about various aspects of the disease that the patient has collected over his/her life, both before and after the diagnosis. These beliefs usually pertain to the causes of the disease and exacerbating factors, identification of symptoms, and available methods of treatments and consequences. These beliefs are collected from different sources, such as stereotypes concerning a given disease, previous personal experiences, medical staff, books, or the Internet. The accuracy of these beliefs may vary and some of them may actually be false. The degree of their correctness can be objectively verified against the current state of medical knowledge about the disease. Knowledge of one's own disease has often been emphasized as an important cognitive factor that can have a considerable impact on the patient's adaptation to the disease and on the course of the disease and its treatment. However, the exact mechanisms through which knowledge of the disease can affect adaptation to the disease are not fully understood. It is possible that there are several pathways on which knowledge of one's own disease can influence psychological adaptation and other health outcomes. One of such pathways may involve the effects on cognitive appraisal.

The concept of cognitive appraisal has been proposed by Lazarus and Folkman [19] to explain the nature of stress processes. Within this framework, cognitive appraisal is treated as a key psychological process mediating the occurrence of a stressor and its outcomes. In fact, Lazarus and Folkman emphasize that cognitive appraisal is not a sum of knowledge about the stressful situation, but it is a more subjective and evaluative process in which the individual attributes subjective meanings to the stressful situation taking into account his/her resources and possible effects of the stressor on well-being. Lazarus and Folkman proposed that three general categories of appraisals (meanings)—Threat, Harm/Loss, and Challenge—are able to instigate the stressful transaction. In this context, disease-related appraisals (DRAs) can be understood as a particular case of the process of cognitive appraisal which refers to the disease as a specific stressor. Therefore, DRAs can be defined as subjective evaluative meanings attributed to one's own disease. Various authors reported there may be more than only three categories of cognitive appraisals related to one's disease [20–22]. These categories are not mutually exclusive but can cooccur, even when they may seem contradictory, which is probably a result of the complex and dynamic nature of disease and stressor [20–22]. The significance of DRAs enforces the fact that they can directly influence the strategies the patient chooses to use to cope with stress of the disease. The effectiveness of coping, in turn, translates into the achieved levels of adaptation to living with a disease. What appraisals and how strongly they are attributed to one's own disease depend on many individual and situational factors; one of them is social support.

Social support can be defined as an external personal resource available to the individual especially when faced with difficult (stressful) situations. It is most commonly conceptualized as perceived social support, which means support subjectively perceived as available, in contrast to received social support, which means the objective amounts of help actually provided by others. In the context of health and disease, social support has often been studied as one of the key variables able to reduce negative effects of stress on health. Since having a chronic disease created its own stress, social support has also been linked to better outcomes of coping with disease-related stress. These positive effects of social support can be attributed to the impact it exerts on the process of coping with stress, and in fact, seeking social support is often considered one of the strategies people employ to cope with disease-related stress. Additionally, social support can contribute to better adaptation to the disease also through its impact on the process of cognitive appraisal. In this case, social support can motivate people to interpret their stressful situation less negatively [23].

The goal of the present study was to verify whether the knowledge of one's own disease and perceived social support are linked to DRAs in women with UI. In particular, we wanted to test which DRAs are correlated with levels of the knowledge of one's own disease and levels of social support. Additionally we also wanted to test whether women with UI with different constellations of the knowledge and social support levels exhibit different patterns of DRAs.

## 2. Methods

### 2.1. Participants

The sample consisted of 158 women treated in an outpatient gynaecological clinic in Nałęczów, Poland, in whom stress urinary incontinence was diagnosed through initial screening. All participants underwent urodynamic tests, on the basis of which the diagnosis was established and severity of the condition was assessed. In the majority of the women, the symptoms of UI had been present over half a year prior to the study and had never been diagnosed or treated. 100% of the patients were women with stress urinary incontinence. The age of the participants ranged from 32 to 79, with the mean age 53.4 (SD = 11,3). A majority of them had an early stage of UI. Participation in the study was voluntary, and written informed consent had been obtained from all the participants before enrolment into the study. The study protocol was approved by the local University Ethical Committee.

### 2.2. Psychological Testing

The participants completed a battery of psychological questionnaires.

*(1) Knowledge of Urinary Incontinence Questionnaire*. It is developed by Szymona-Pałkowska and Kraczkowski to measure the patients' levels of knowledge of UI. This method consists of 25 statements regarding UI, its causes, predisposing and exacerbating factors, and methods of treatment. The statements were generated on the basis of relevant literature and pilot studies carried out in the city hospitals in Lublin in the years 2008-2009. The respondents are requested to judge whether a given statement is true (true, do not know, and false). The possible scores range between 0 and 25, with higher scores denoting higher levels of knowledge [24, 25].

*(2) Disease-Related Social Support Scale.* It consists of 30 items in the form of statements describing different types of social support which can be perceived by the patient over the course of the disease. The participants respond to each statement on a 4-point scale. The scores correspond to five types of social support: emotional, material, spiritual, instrumental, and informational support. The total score from all items is a global index of perceived social support, where higher values indicate higher perceived social support. The internal consistency reliabilities (Cronbach's *α*) for the measure are high and very high, ranging from 0.84 for material support to 0.94 for the total score [26].

*(3) Disease-Related Appraisals Scale.* It is a multidimensional self-report questionnaire designed to measure subjective meanings patients may attribute to their disease. It consists of 47 items that make up 7 subscales: six appraisals—Threat, Profit, Obstacle/Loss, Challenge, Harm, and Value—and one control subscale labelled Importance, measuring overall importance given to the disease. Each item is assessed on a 5-grade scale. Higher scores indicate a stronger appraisal of the disease within a given category. The scale is characterized by satisfactory and high reliability coefficients, and Cronbach's alphas for the subscales range from *α* = 0.64 for the Challenge to *α* = 0.87 for Obstacle/Loss [22].

### 2.3. Statistical Analyses

Pearson's *r* correlation coefficients were calculated to analyse the relationships between the disease-related appraisals, on one hand, and the patients' levels of the knowledge of UI and social support, on the other hand. In the next step of the analysis, the patients scores on the global index of social support and levels of the knowledge were linearly transformed into standardized *z*-scores. These scores were entered into hierarchical cluster analysis (*k*-means method) to divide the patients into subgroups with different constellations of the knowledge and social support. The obtained subgroups were later compared with regard to disease-related appraisals using one-way ANOVA. The Lowest Significant Difference (LSD) test was used for post hoc analysis.

## 3. Results

### 3.1. Knowledge of One's Disease, Social Support, and Disease-Related Appraisals: Correlational Analyses

The level of the knowledge correlated significantly negatively with the appraisal of the disease as Harm (*r* = −0.24, *P* ≤ 0.01). No statistically significant correlations were found between the knowledge and the remaining disease-related appraisals. The global level of social support correlated significantly positively with three disease-related appraisals—Profit (*r* = 0.29, *P* ≤ 0.01), Challenge (*r* = 0.31, *P* ≤ 0.001), and Value (*r* = 0.25, *P* ≤ 0.01). Additionally, two subtypes of social support showed specific correlations. Namely, instrumental support correlated significantly positively with the disease appraisal of Threat and informational support correlated significantly positively with the appraisals of Threat and Obstacle/Loss. Informational support also correlated significantly positively with overall importance attributed to the disease. The detailed matrix of the obtained correlations is presented in Table 1.

### 3.2. Disease-Related Appraisals in Subgroups of Women with Different Constellations of Knowledge and Social Support

**Figure 1:**
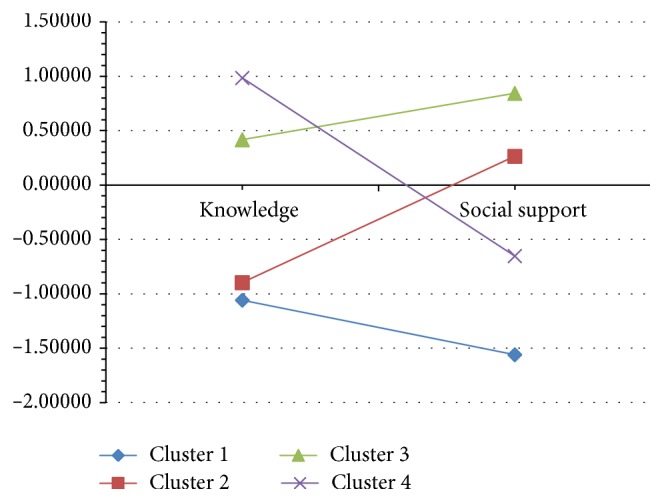
Mean standardized *z*-scores on knowledge of UI and global social support in four subgroups of patients obtained in hierarchical cluster analysis.

The patients' scores on the global index of social support and on the knowledge of UI were transformed into standardized *z*-scores and taken as criterion variables to hierarchical cluster analyses to yield subgroups of patients with different constellations of these variables. The four-cluster solution was selected. The resultant mean scores on the knowledge and social support within the four obtained subgroups of patients are presented in Table 2 and Figure 1.

The four distinguished subgroups of women with UI were compared with regard to the scores on disease-related appraisals using one-way ANOVA. The results of this analysis are presented in Table 3. Most women (*N* = 57) were classified as those with both high knowledge and high social support (Cluster 3). Forty women were classified as presenting low levels of the knowledge but high support (Cluster 2). Thirty-five patients were classified as possessing high knowledge but low social support (Cluster 4). Twenty-three women were classified as those with low knowledge and low support (Cluster 1).

**Figure 2:**
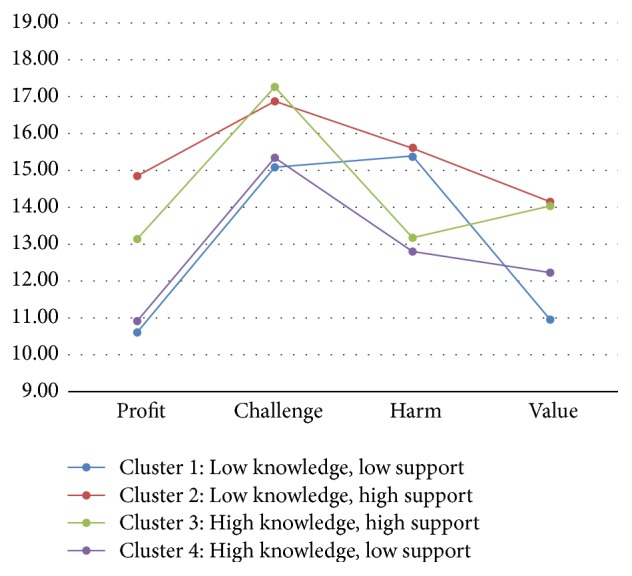
Mean scores on the appraisals of Profit, Challenge, Harm, and Value in four subgroups of patients identified in hierarchical cluster analysis.

The subgroups of patients with different constellations of the knowledge of UI and social support were found to differ statistically significantly with regard to four disease-related appraisals: Profit, Challenge, Harm, and Value. The profiles of the scores on these appraisals for the subgroups are presented graphically in Figure 2.

Two subgroups with high levels of social support (Cluster 2 and Cluster 3) scored significantly higher than the subgroups with low social support (Cluster 1 and Cluster 4) on all positive disease-related appraisals (Profit, Challenge, and Value) (LSD post hoc tests: *P* < 0.05). However, the subgroup with high social support and high knowledge (Cluster 3) scored significantly lower (*P* < 0.5) on the disease-related appraisal of Harm than the subgroup with high social support but low levels of the knowledge (Cluster 2). Two subgroups with low social support (Cluster 1 and Cluster 4) were very similar (*P* > 0.5) with regard to their low scores on positive disease-related appraisals (Profit, Challenge, and Value). They differed significantly on the negative appraisal of Harm, with the subgroup with low support but high knowledge (Cluster 4) scoring significantly lower (*P* < 0.5) than the subgroup with both low support and low knowledge (Cluster 1).

## 4. Discussion

Patients derive the knowledge of their disease from various sources, for example, information from physicians or other health care professionals, using other sufferers' opinions, information available in mass media, scientific journals, or the Internet forums. This knowledge can have varying degrees of accuracy, when confronted against the current state of objective medical knowledge. It is usually believed that patients who are better educated about their disease and who have more accurate knowledge of their disease will comply better with the treatment [27, 28] and show better overall psychological adaptation to the disease [29, 30]. In contrast, incomplete or inaccurate knowledge of one's disease may be a limiting factor in treatment [31]. These positive outcomes of accurate knowledge of one's disease are often explained by the effects knowledge may exert on the patients' emotional states, especially by reducing anxiety [32] and increasing the sense of controllability and predictability [33]. In our study we wanted to test whether the levels of the knowledge female patients with UI reveal about their disease could affect adaptation through the impact on disease-related appraisals. In our study we used an objective measure of the patients' knowledge of UI to determine the levels of accurate up-to-date disease-related knowledge. Our results demonstrated that the effects of the knowledge of UI on disease-related appraisals are limited to one appraisal, namely, Harm. The levels of the knowledge correlated significantly negatively with this category of appraisal, suggesting that patients with higher knowledge of UI tend to perceive less in terms of Harm. However, no significant correlations were observed between the level of the knowledge and Threat. This may suggest that accurate knowledge may operate more through reducing sense of guilt, shame, or injustice related to the fact of being ill (as derivatives of the appraisal of Harm), rather than by reducing anxiety (as a derivative of the appraisal of Threat). Whether this effect is universal across more clinical populations or specific to patients with UI requires verification in further research.

Social support is an important psychosocial resource which provides patients with psychological aid, information, or advice and whose role is to help one to find a solution and facilitate contacts with relevant institutions. Social support can also help to instruct how to use aid and how to satisfy the need of being consoled and heard or improve one's financial status [34]. It is a way to reach useful or essential information with the help of other people [35]. In our study we also wanted to analyse the possible links between social support and disease-related appraisals in women with UI. The correlational analysis we performed demonstrated that global levels of social support the women perceived as available to them over the period of being ill were positively associated with three positive appraisals: Profit, Challenge, and Value, and remained unrelated to the negative appraisals of Threat, Obstacle/Loss, or Harm (even though some subtypes of support may have correlated with Threat or Obstacle/Loss). This finding emphasizes the favourable role of social support in adaptation to the disease in patients with UI. Positive disease-related appraisals have been linked to better adaptational outcomes, such as higher quality of life, lower depressive symptoms, or higher acceptance of life with disease, in previous research carried out among patients with UI [36], low back pain [37], and psoriasis [38]. Additionally, the findings from this study demonstrating the links between social support and positive disease-related appraisals provide arguments for the hypothesis that social support operates by inducing the patients' positive (salutogenic) resources rather than reducing negative (pathogenic) factors.

In our next analysis, we classified the patients into four subgroups (clusters) with different constellations of the knowledge of UI and social support. These subgroups were found to differ significantly statistically with respect to the appraisals of Profit, Challenge, Harm, and Value. Even a superficial analysis of the profiles (Figure 2) within the subgroups can reveal that the subgroup with high levels of both social support and knowledge (Cluster 3) exhibited the most adaptive pattern of appraisals (high appraisals of Challenge and Value, average appraisal of Profit, and low appraisal of Harm). In contrast, the subgroup with low levels of both social support and knowledge (Cluster 1) revealed the least favourable pattern of appraisals (low appraisals of Profit, Challenge, and Value, while high appraisal of Harm). This suggests that both knowledge of UI and social support contribute to the way the patients appraise their disease. However, certain differences in this contribution between social support and knowledge may be indicated. Social support seems to have more effect on the positive disease-related appraisals of Profit, Challenge, and Value, whereas the knowledge of UI exerts and has effects on the negative appraisal of Harm. This is evident in the fact that the subgroups with high social support were similar with regard to levels of positive appraisals irrespective of the level of knowledge. However, they differed significantly with respect to the appraisal of Harm. The exactly opposite pattern was observed for the subgroups with low levels of support.

Generally, our findings indicate that scant knowledge and little social support both interact to contribute to an unfavourable pattern of disease-related appraisals in women with UI. Women with low knowledge and low social support are less likely to appraise their UI condition positively and they are overwhelmed by the sense of Harm. On the other hand, high knowledge of UI and high social support both interact to promote the most adaptive pattern of disease-related appraisals. Our research indicates that an increased knowledge can decrease the women's tendency to perceive their condition in terms of Harm. Accurate knowledge of UI and high levels of social support probably provide optimal circumstances, which help the women with UI maintain the most adaptive cognitive interpretation of their condition.

Meanings attributed to the disease are modified by the changing life circumstances, earlier experiences, and social support, and they may affect the sufferers' more global attitudes, affects, and behaviors towards their symptoms and treatment recommendations. Cognitive appraisal of an illness may affect the whole process of adaptation and resultant quality of life [39].

## Figures and Tables

**Table 1:** Pearson's *r* correlation coefficients between the patients' level of knowledge (*Knowledge of Urinary Incontinence Questionnaire*), social support (*Disease-Related Social Support Scale*), and disease-related appraisals (*Disease-Related Appraisals Scale*).

	Threat	Profit	Obstacle/Loss	Challenge	Harm	Value	Importance
Knowledge	−0.07	−0.11	−0.07	−0.05	−0.24^*∗∗*^	−0.04	0.05
Social support: global score	0.15	0.29^*∗∗∗*^	0.10	0.31^*∗∗∗*^	−0.12	0.25^*∗∗*^	−0.01
Spiritual support	0.06	0.26^*∗∗∗*^	0.02	0.29^*∗∗∗*^	−0.12	0.34^*∗∗∗*^	−0.13
Instrumental support	0.17^*∗*^	0.24^*∗∗*^	0.11	0.26^*∗∗∗*^	−0.11	0.10	0.05
Informational support	0.20^*∗*^	0.20^*∗*^	0.18^*∗*^	0.29^*∗∗∗*^	−0.07	0.16	0.19^*∗*^
Material support	0.12	0.28^*∗∗∗*^	0.12	0.25^*∗∗*^	−0.10	0.20^*∗*^	0.02
Emotional support	0.12	0.31^*∗∗∗*^	0.03	0.27^*∗∗∗*^	−0.12	0.30^*∗∗∗*^	−0.09

^*∗∗*^ *P* ≤ 0.01. ^*∗*^ and ^*∗∗∗*^ show level of correlation.

**Table 2:** Mean standardized *z*-scores on knowledge of UI and global social support in four subgroups of patients obtained in hierarchical cluster analysis.

	Cluster 1	Cluster 2	Cluster 3	Cluster 4
	Low knowledge, very low support	Low knowledge, moderate support	High knowledge, high support	High knowledge, low support
	*N* = 23	*N* = 40	*N* = 57	*N* = 35
	*M*	*M*	*M*	*M*
Knowledge (*z*-scores)	−1.06	−0.90	0.42	0.99
Global social support (*z*-scores)	−1.56	0.26	0.84	−0.65

**Table 3:** Mean values and standard deviations for disease-related appraisals in subgroups of women identified in cluster analysis.

	Cluster 1		Cluster 2		Cluster 3		Cluster 4		ANOVA	
	Low knowledge, low support		Low knowledge, high support		High knowledge, high support		High knowledge, low support			
	*N* = 23		*N* = 40		*N* = 57		*N* = 35			
	*M*	SD	*M*	SD	*M*	SD	*M*	SD	*F*	*P*
Threat	21.65	6.85	23.85	5.39	22.61	5.23	20.51	6.99	2.09	0.103
Profit	10.61	2.62	14.85	4.75	13.14	3.67	10.91	2.93	9.89	0.000
Obstacle	17.65	7.44	20.80	5.50	18.60	5.23	18.57	6.68	1.73	0.164
Challenge	15.09	4.46	16.88	3.62	17.26	3.41	15.34	4.30	2.94	0.035
Harm	15.39	7.22	15.60	4.70	13.18	4.78	12.80	4.69	2.94	0.035
Value	10.96	2.79	14.15	4.35	14.04	4.27	12.23	4.75	4.19	0.007
Importance	14.55	3.19	14.87	2.63	14.64	4.79	14.79	3.30	0.04	0.987
